# The genome sequence of the King Ragworm, *Alitta virens *(Sars, 1835)

**DOI:** 10.12688/wellcomeopenres.19642.1

**Published:** 2023-07-11

**Authors:** Chris Fletcher, Lyndall Pereira da Conceicoa, Natural History Museum Genome Acquisition Lab, Darwin Tree of Life Barcoding collective, Wellcome Sanger Institute Tree of Life programme, Wellcome Sanger Institute Scientific Operations: DNA Pipelines collective, Tree of Life Core Informatics collective, Darwin Tree of Life Consortium

**Affiliations:** 1 Natural History Museum, London, England, UK; 2 Wellcome Sanger Institute, Hinxton, England, UK

**Keywords:** Alitta virens, King Ragworm, genome sequence, chromosomal, Phyllodocida

## Abstract

We present a genome assembly from an individual *Alitta virens *(the King Ragworm; Annelida; Polychaeta; Phyllodocida; Nereididae). The genome sequence is 671.2 megabases in span. Most of the assembly is scaffolded into 14 chromosomal pseudomolecules. The mitochondrial genome has also been assembled and is 15.83 kilobases in length.

## Species taxonomy

Eukaryota; Opisthokonta; Metazoa; Eumetazoa; Bilateria; Protostomia; Spiralia; Lophotrochozoa; Annelida; Polychaeta; Errantia; Phyllodocida; Nereididae; Alitta (Sars, 1835) (NCBI:txid880429).

## Background

*Alitta virens*, commonly known as the King Ragworm, is a polychaete species from the family Nereididae. It is one of the larger intertidal polychaetes, reported by Chambers and Garwood (1992) as reaching up to 320 mm long with 160 chaetigers. It is common in sheltered muddy and sandy intertidal zones and estuaries throughout the UK and Europe ( Chambers & Garwood, 1992; Kristensen, 1984). *A. virens* burrows into the sediment, feeding using a combination of deposit feeding and suspension feeding, the latter by using mucus nets ( Herringshaw *et al.*, 2010).

Reproduction most likely occurs in the same way as other ragworms such as *Hediste diversicolor*, where females release oocytes from their burrows to be fertilised by males. Larvae will settle in the upper intertidal zone and migrate to lower intertidal zone after approximately 3 years, but this may vary depending on habitat and size of the individual ( Desrosiers *et al.*, 1994; Kristensen, 1984).

*A .virens* preys on other invertebrate species but is also an important prey species for birds, fish and other crustaceans. Along with other species of large intertidal polychaete, it is also widely commercially and recreationally exploited worldwide, owing to its popularity as bait for recreational fishing. ( Devon & Severn IFCA, 2019; Olive, 1994; Watson *et al.*, 2017)

Here we present the chromosomally complete genome sequence for *A. virens*, sequenced as part of the Darwin Tree of Life Project. It is hoped that this will provide further insight into the biology, ecology and evolution of *A. virens* and other nereid worms.

## Genome sequence report

**Figure 1.**
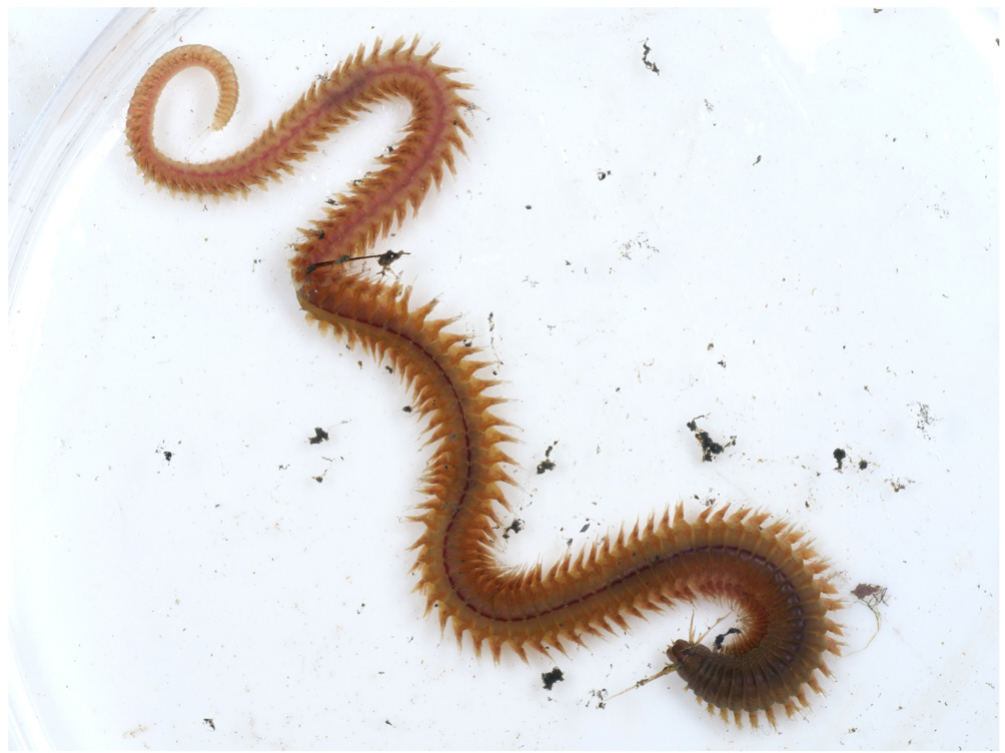
Photograph of the *Alitta virens* (wpAliVire1) specimen used for genome sequencing.

The genome was sequenced from one *Alitta virens* ( Figure 1) collected from Newtown Quay Lagoon, England (50.72, –1.41). A total of 49-fold coverage in Pacific Biosciences single-molecule HiFi long reads was generated. Primary assembly contigs were scaffolded with chromosome conformation Hi-C data. Manual assembly curation corrected 143 missing joins or mis-joins and removed 7 haplotypic duplications, reducing the assembly length by 0.23% and the scaffold number by 73.03%, and increasing the scaffold N50 by 6.68%.

**Figure 2.**
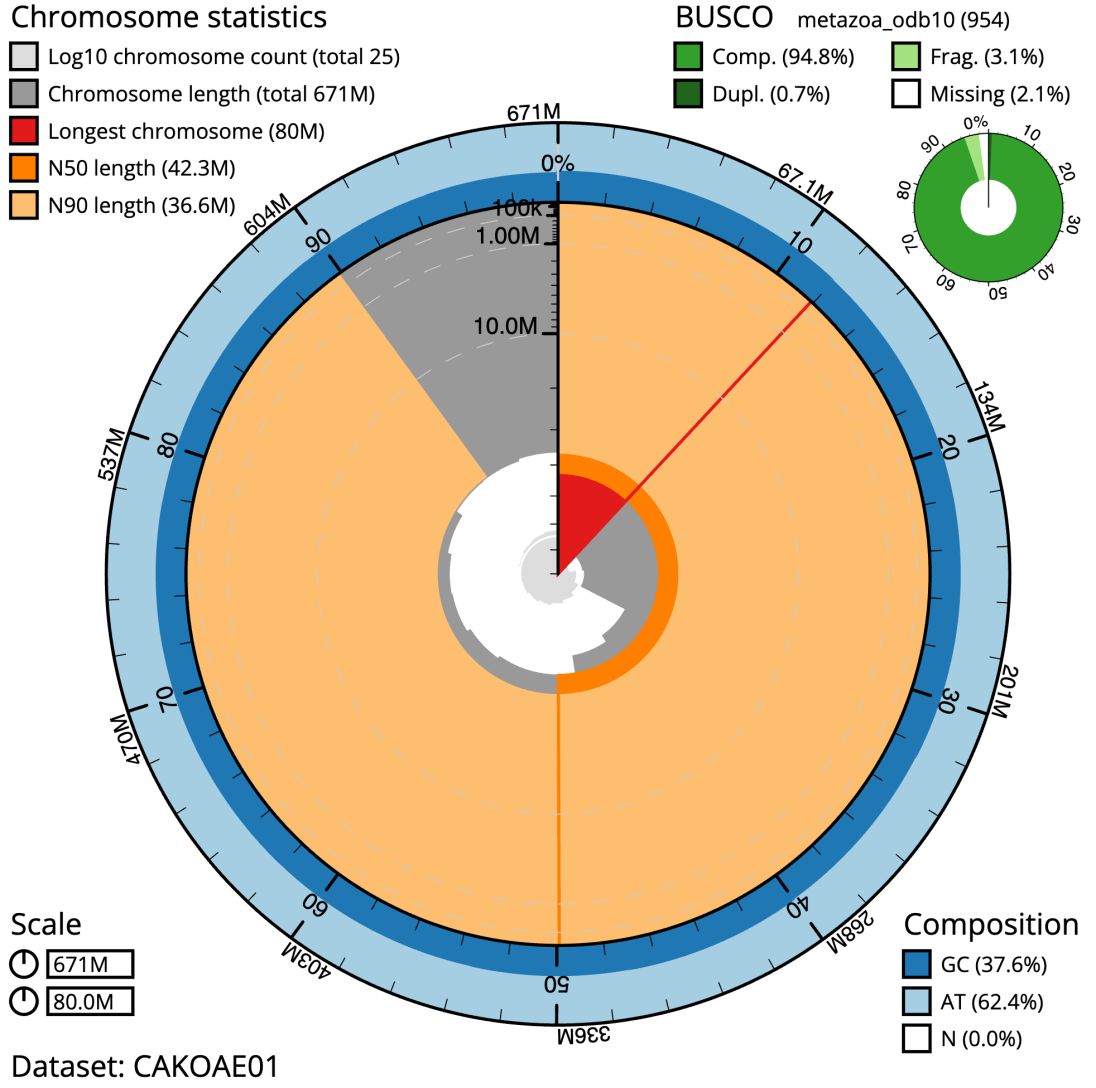
Genome assembly of *Alitta virens*, wpAliVire1.1: metrics. The BlobToolKit Snailplot shows N50 metrics and BUSCO gene completeness. The main plot is divided into 1,000 size-ordered bins around the circumference with each bin representing 0.1% of the 671,169,925 bp assembly. The distribution of scaffold lengths is shown in dark grey with the plot radius scaled to the longest scaffold present in the assembly (80,028,313 bp, shown in red). Orange and pale-orange arcs show the N50 and N90 scaffold lengths (42,251,612 and 36,633,593 bp), respectively. The pale grey spiral shows the cumulative scaffold count on a log scale with white scale lines showing successive orders of magnitude. The blue and pale-blue area around the outside of the plot shows the distribution of GC, AT and N percentages in the same bins as the inner plot. A summary of complete, fragmented, duplicated and missing BUSCO genes in the metazoa_odb10 set is shown in the top right. An interactive version of this figure is available at https://blobtoolkit.genomehubs.org/view/wpAliVire1.1/dataset/CAKOAE01/snail.

The final assembly has a total length of 671.2 Mb in 24 sequence scaffolds with a scaffold N50 of 42.3 Mb ( Table 1). Most (99.97%) of the assembly sequence was assigned to 14 chromosomal-level scaffolds. Chromosome-scale scaffolds confirmed by the Hi-C data are named in order of size ( Figure 2– Figure 5; Table 2). Repetitive scaffolds have been inserted into a repeat region of chromosome 4, but their order and orientation is uncertain. While not fully phased, the assembly deposited is of one haplotype. Contigs corresponding to the second haplotype have also been deposited. The mitochondrial genome was also assembled and can be found as a contig within the multifasta file of the genome submission.

**Table 1.** Genome data for *Alitta virens*, wpAliVire1.1.

Project accession data		
Assembly identifier	wpAliVire1.1	
Species	*Alitta virens*	
Specimen	wpAliVire1	
NCBI taxonomy ID	880429	
BioProject	PRJEB50793	
BioSample ID	SAMEA8534434	
Isolate information	wpAliVire1, posterior body (DNA sequencing, Hi-C scaffolding, RNA sequencing)	
Assembly metrics *		*Benchmark*
Consensus quality (QV)	62.6	*≥ 50*
*k*-mer completeness	100%	*≥ 95%*
BUSCO **	C:94.8%[S:94.0%,D:0.7%], F:3.1%,M:2.1%,n:954	*C ≥ 95%*
Percentage of assembly mapped to chromosomes	99.97%	*≥ 95%*
Sex chromosomes	-	*localised homologous pairs*
Organelles	Mitochondrial genome assembled	*complete single alleles*
Raw data accessions		
PacificBiosciences SEQUEL II	ERR8575399, ERR8575400	
Hi-C Illumina	ERR8571700	
PolyA RNA-Seq Illumina	ERR10123675	
Genome assembly		
Assembly accession	GCA_932294295.1	
*Accession of alternate haplotype*	GCA_932294305.1	
Span (Mb)	671.2	
Number of contigs	219	
Contig N50 length (Mb)	7.8	
Number of scaffolds	24	
Scaffold N50 length (Mb)	42.3	
Longest scaffold (Mb)	80.0	

* Assembly metric benchmarks are adapted from column VGP-2020 of “Table 1: Proposed standards and metrics for defining genome assembly quality” from ( Rhie *et al.*, 2021). ** BUSCO scores based on the metazoa_odb10 BUSCO set using v5.3.2. C = complete [S = single copy, D = duplicated], F = fragmented, M = missing, n = number of orthologues in comparison. A full set of BUSCO scores is available at https://blobtoolkit.genomehubs.org/view/wpAliVire1.1/dataset/CAKOAE01/busco.

**Figure 3.**
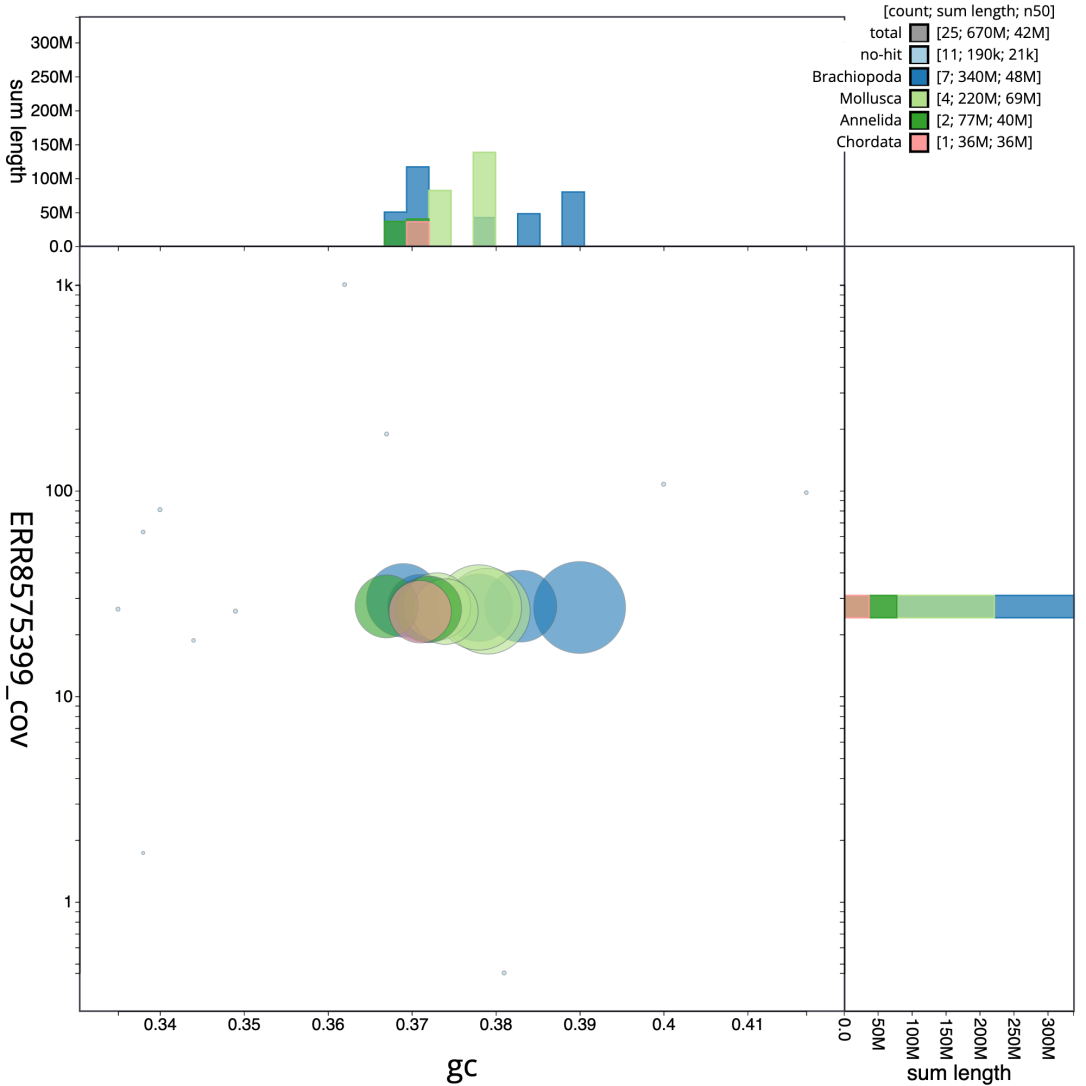
Genome assembly of *Alitta virens*, wpAliVire1.1: BlobToolKit GC-coverage plot. Scaffolds are coloured by phylum. Circles are sized in proportion to scaffold length. Histograms show the distribution of scaffold length sum along each axis. An interactive version of this figure is available at https://blobtoolkit.genomehubs.org/view/wpAliVire1.1/dataset/CAKOAE01/blob.

**Figure 4.**
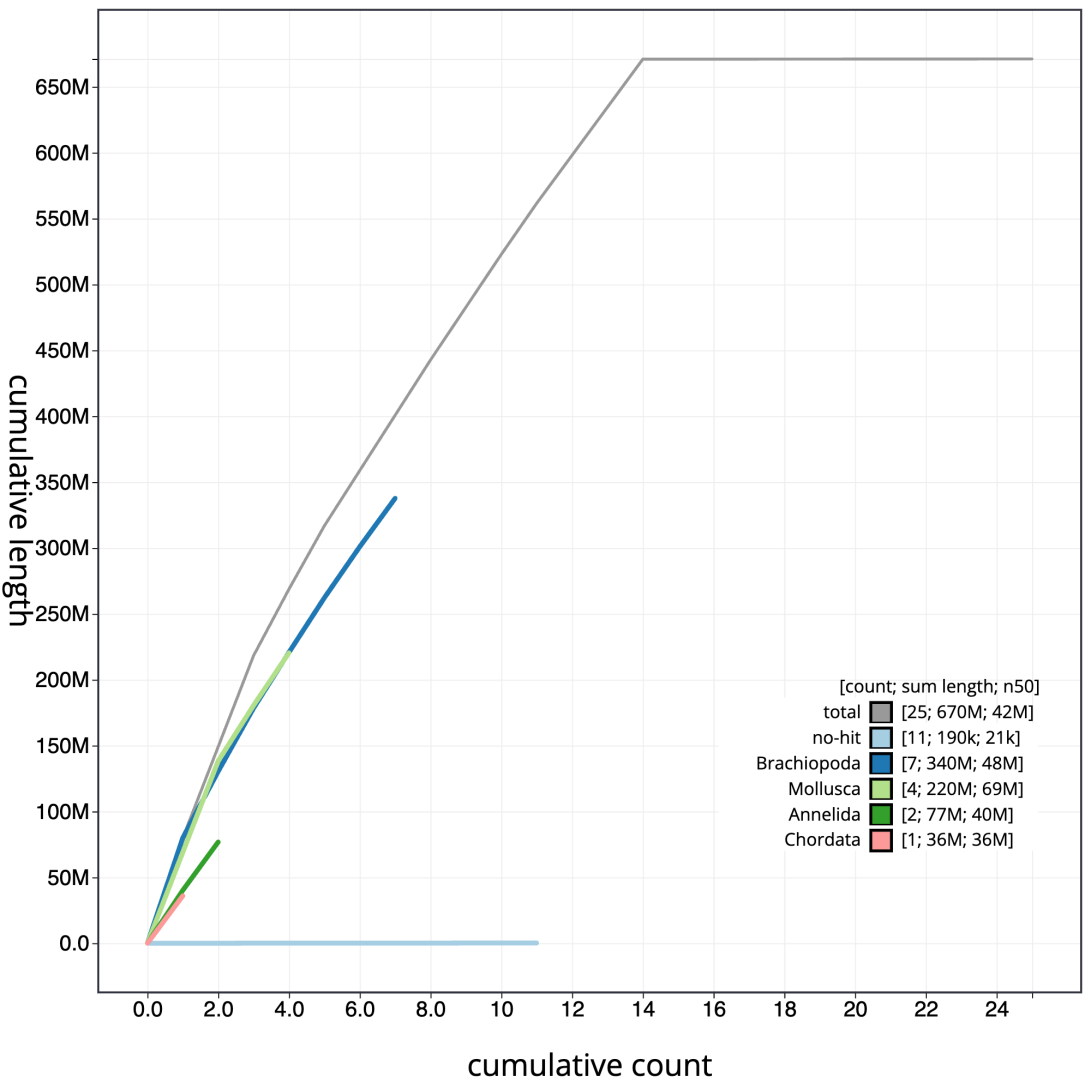
Genome assembly of *Alitta virens*, wpAliVire1.1: BlobToolKit cumulative sequence plot. The grey line shows cumulative length for all scaffolds. Coloured lines show cumulative lengths of scaffolds assigned to each phylum using the buscogenes taxrule. An interactive version of this figure is available at https://blobtoolkit.genomehubs.org/view/wpAliVire1.1/dataset/CAKOAE01/cumulative.

**Figure 5.**
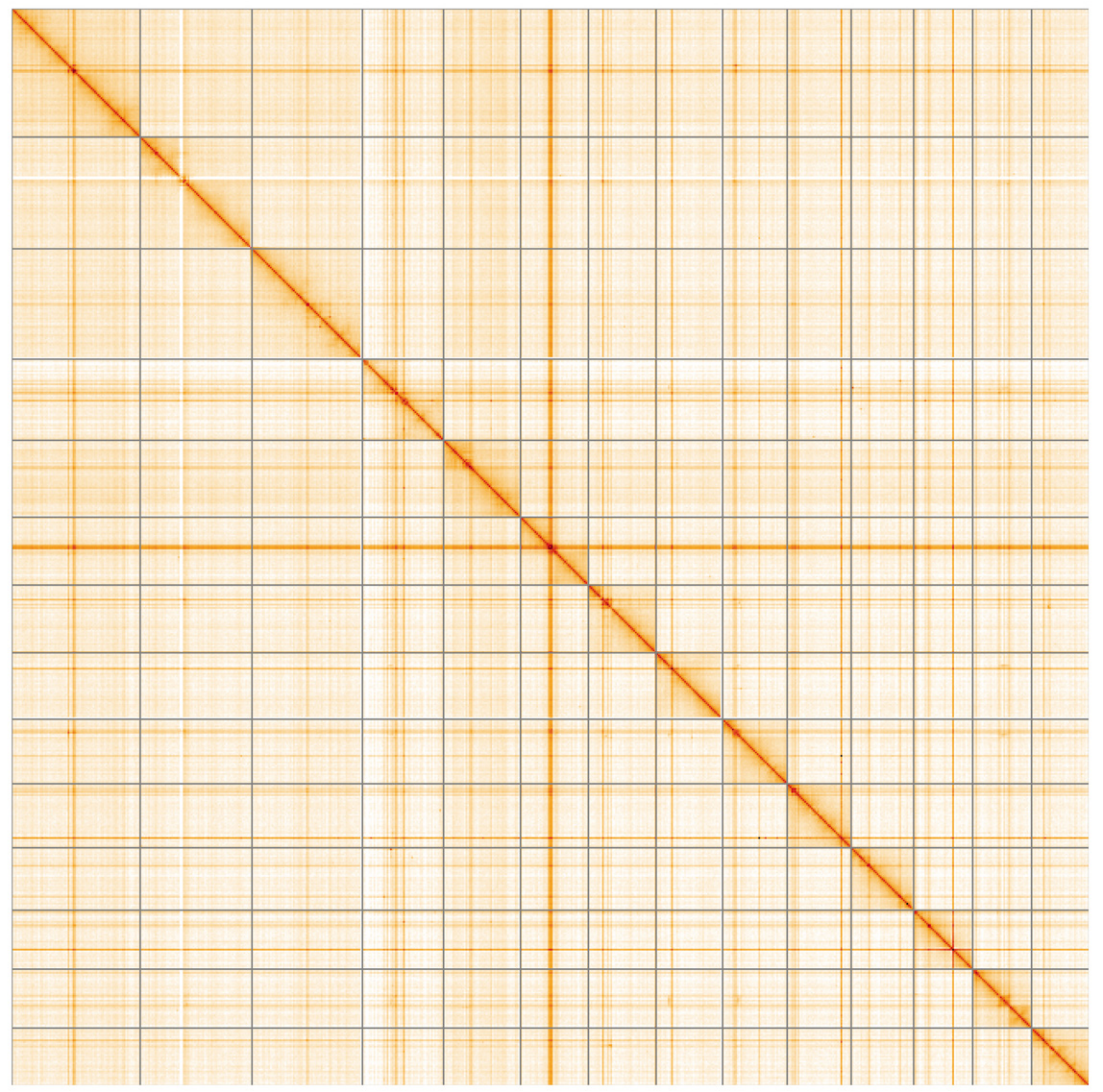
Genome assembly of *Alitta virens*, wpAliVire1.1: Hi-C contact map of the wpAliVire1.1 assembly, visualised using HiGlass. Chromosomes are shown in order of size from left to right and top to bottom. An interactive version of this figure may be viewed at https://genome-note-higlass.tol.sanger.ac.uk/l/?d=IlOLYG6YRWODiRYfatsIpA.

**Table 2.** Chromosomal pseudomolecules in the genome assembly of *Alitta virens*, wpAliVire1.

INSDC accession	Chromosome	Length (Mb)	GC%
OW028573.1	1	80.03	39.0
OW028574.1	2	69.45	38.0
OW028575.1	3	68.83	38.0
OW028576.1	4	50.47	37.0
OW028577.1	5	48.02	38.5
OW028578.1	6	42.25	38.0
OW028579.1	7	42.24	37.5
OW028580.1	8	41.4	37.0
OW028581.1	9	40.21	37.0
OW028582.1	10	39.91	37.5
OW028583.1	11	38.77	37.0
OW028584.1	12	36.77	37.0
OW028585.1	13	36.63	36.5
OW028586.1	14	36.01	37.0
OW028587.1	MT	0.02	36.0

The estimated Quality Value (QV) of the final assembly is 62.6 with *k*-mer completeness of 100%, and the assembly has a BUSCO v5.3.2 completeness of 94.8% (single = 94.0%, duplicated = 0.7%), using the metazoa_odb10 reference set ( *n* = 954).

Metadata for specimens, spectral estimates, sequencing runs, contaminants and pre-curation assembly statistics can be found at https://links.tol.sanger.ac.uk/species/880429.

## Methods

### Sample acquisition and nucleic acid extraction

An *Alitta virens* specimen (wpAliVire1) was collected from Newtown Quay Lagoon, UK (latitude 50.72, longitude –1.41) on 2020-10-11 using a pond net. The specimen was collected by Chris Fletcher (Natural History Museum) and Lyndall Pereira Da Conceicoa (Wellcome Sanger Institute) and identified by Chris Fletcher. Body tissue extracted from the specimen was preserved in liquid nitrogen. The remainder of the specimen was preserved in 80% ethanol and stored at the Natural History Museum, London.

DNA was extracted at the Tree of Life laboratory, Wellcome Sanger Institute (WSI). The wpAliVire1 sample was weighed and dissected on dry ice with tissue set aside for Hi-C sequencing. Posterior body tissue was disrupted using a Nippi Powermasher fitted with a BioMasher pestle. High molecular weight (HMW) DNA was extracted using the Qiagen MagAttract HMW DNA extraction kit. HMW DNA was sheared into an average fragment size of 12–20 kb in a Megaruptor 3 system with speed setting 30. Sheared DNA was purified by solid-phase reversible immobilisation using AMPure PB beads with a 1.8X ratio of beads to sample to remove the shorter fragments and concentrate the DNA sample. The concentration of the sheared and purified DNA was assessed using a Nanodrop spectrophotometer and Qubit Fluorometer and Qubit dsDNA High Sensitivity Assay kit. Fragment size distribution was evaluated by running the sample on the FemtoPulse system.

RNA was extracted from tissue of wpAliVire1 in the Tree of Life Laboratory at the WSI using TRIzol, according to the manufacturer’s instructions. RNA was then eluted in 50 μl RNAse-free water and its concentration assessed using a Nanodrop spectrophotometer and Qubit Fluorometer using the Qubit RNA Broad-Range (BR) Assay kit. Analysis of the integrity of the RNA was done using Agilent RNA 6000 Pico Kit and Eukaryotic Total RNA assay.

### Sequencing

Pacific Biosciences HiFi circular consensus DNA sequencing libraries were constructed according to the manufacturers’ instructions. Poly(A) RNA-Seq libraries were constructed using the NEB Ultra II RNA Library Prep kit. DNA and RNA sequencing were performed by the Scientific Operations core at the WSI on Pacific Biosciences SEQUEL II (HiFi) and Illumina NovaSeq 6000 (RNA-Seq) instruments. Hi-C data were also generated from tissue of wpAliVire1 using the Arimav2 kit and sequenced on the Illumina NovaSeq 6000 instrument.

### Genome assembly, curation and evaluation

Assembly was carried out with Hifiasm ( Cheng *et al.*, 2021) and haplotypic duplication was identified and removed with purge_dups ( Guan *et al.*, 2020). The assembly was scaffolded with Hi-C data ( Rao *et al.*, 2014) using YaHS ( Zhou *et al.*, 2023). The assembly was checked for contamination and corrected as described previously ( Howe *et al.*, 2021). Manual curation was performed using HiGlass ( Kerpedjiev *et al.*, 2018) and Pretext ( Harry, 2022). The mitochondrial genome was assembled using MitoHiFi ( Uliano-Silva *et al.*, 2022), which runs MitoFinder ( Allio *et al.*, 2020) or MITOS ( Bernt *et al.*, 2013) and uses these annotations to select the final mitochondrial contig and to ensure the general quality of the sequence.

A Hi-C map for the final assembly was produced using bwa-mem2 ( Vasimuddin *et al.*, 2019) in the Cooler file format ( Abdennur & Mirny, 2020). To assess the assembly metrics, the *k*-mer completeness and QV consensus quality values were calculated in Merqury ( Rhie *et al.*, 2020). This work was done using Nextflow ( Di Tommaso *et al.*, 2017) DSL2 pipelines “sanger-tol/readmapping” ( Surana *et al.*, 2023a) and “sanger-tol/genomenote” ( Surana *et al.*, 2023b). The genome was analysed within the BlobToolKit environment ( Challis *et al.*, 2020) and BUSCO scores ( Manni *et al.*, 2021; Simão *et al.*, 2015) were calculated.

Table 3 contains a list of relevant software tool versions and sources.

**Table 3.** Software tools: versions and sources.

Software tool	Version	Source
BlobToolKit	4.1.5	https://github.com/blobtoolkit/ blobtoolkit
BUSCO	5.3.2	https://gitlab.com/ezlab/busco
Hifiasm	0.16.1-r375	https://github.com/chhylp123/ hifiasm
HiGlass	1.11.6	https://github.com/higlass/ higlass
Merqury	MerquryFK	https://github.com/ thegenemyers/MERQURY.FK
MitoHiFi	2	https://github.com/ marcelauliano/MitoHiFi
PretextView	0.2	https://github.com/wtsi-hpag/ PretextView
purge_dups	1.2.3	https://github.com/dfguan/ purge_dups
sanger-tol/ genomenote	v1.0	https://github.com/sanger-tol/ genomenote
sanger-tol/ readmapping	1.1.0	https://github.com/sanger-tol/ readmapping/tree/1.1.0
YaHS	yahs- 1.1.91eebc2	https://github.com/c-zhou/yahs

### Wellcome Sanger Institute – Legal and Governance

The materials that have contributed to this genome note have been supplied by a Darwin Tree of Life Partner. The submission of materials by a Darwin Tree of Life Partner is subject to the **‘Darwin Tree of Life Project Sampling Code of Practice’**, which can be found in full on the Darwin Tree of Life website here. By agreeing with and signing up to the Sampling Code of Practice, the Darwin Tree of Life Partner agrees they will meet the legal and ethical requirements and standards set out within this document in respect of all samples acquired for, and supplied to, the Darwin Tree of Life Project.

Further, the Wellcome Sanger Institute employs a process whereby due diligence is carried out proportionate to the nature of the materials themselves, and the circumstances under which they have been/are to be collected and provided for use. The purpose of this is to address and mitigate any potential legal and/or ethical implications of receipt and use of the materials as part of the research project, and to ensure that in doing so we align with best practice wherever possible. The overarching areas of consideration are:

- Ethical review of provenance and sourcing of the material
- Legality of collection, transfer and use (national and international)

Each transfer of samples is further undertaken according to a Research Collaboration Agreement or Material Transfer Agreement entered into by the Darwin Tree of Life Partner, Genome Research Limited (operating as the Wellcome Sanger Institute), and in some circumstances other Darwin Tree of Life collaborators.

## Data Availability

European Nucleotide Archive: *Alitta virens*. Accession number PRJEB50793; https://identifiers.org/ena.embl/PRJEB50793. ( Wellcome Sanger Institute, 2022) The genome sequence is released openly for reuse. The *Alitta virens* genome sequencing initiative is part of the Darwin Tree of Life (DToL) project. All raw sequence data and the assembly have been deposited in INSDC databases. The genome will be annotated using available RNA-Seq data and presented through the Ensembl pipeline at the European Bioinformatics Institute. Raw data and assembly accession identifiers are reported in Table 1.

## Author information

Members of the Natural History Museum Genome Acquisition Lab are listed here: https://doi.org/10.5281/zenodo.4790042.

Members of the Darwin Tree of Life Barcoding collective are listed here: https://doi.org/10.5281/zenodo.4893703.

Members of the Wellcome Sanger Institute Tree of Life programme are listed here: https://doi.org/10.5281/zenodo.4783585.

Members of Wellcome Sanger Institute Scientific Operations: DNA Pipelines collective are listed here: https://doi.org/10.5281/zenodo.4790455.

Members of the Tree of Life Core Informatics collective are listed here: https://doi.org/10.5281/zenodo.5013541.

Members of the Darwin Tree of Life Consortium are listed here: https://doi.org/10.5281/zenodo.4783558.
