# Supplementary material for: The genome sequence of the King Ragworm, Alitta virens (Sars, 1835)
Source: Wellcome Open Res. 2023 Jul 11;8:297. [Version 1] doi: 10.12688/wellcomeopenres.19642.1 (PMC10352625; doi:10.12688/wellcomeopenres.19642.1)
Supplement: Peer review history

# Peer review: Reviewer response for version 1

**DOI:** 10.21956/wellcomeopenres.21759.r62732

**Published:** 2023-08-18

**Authors:** Ferdinand Marlétaz

**Affiliations:** 1 Centre for Life’s Origin & Evolution, Department of Ecology, Evolution & Environment, University College London, London, UK

This chromosome-scale genome of the king ragworm - an iconic species - is a great resource, and also represent a major achievement as the first nereid genome sequenced so far. The methodology used to assemble seems up to the highest standards. 

**Minor comments:**

 I don't belive the contig N50 out of the Hifiasm has been specified, I would be curious as some polychaete have sometimes been notoriously difficult to assemble at this stage.

 Also I think that it should be specified which tissue was used for RNA-seq and HiC, this is a big worm, with some hard parts and it would be crucial information for the community.

 Similarly, no indications are given of the protocols used for Hi-C: "Hi-C data were also generated from tissue of wpAliVire1 using the Arimav2 kit and sequenced on the Illumina NovaSeq 6000 instrument." Enough detail should be given. 

 It would also be great to reference previous estimate of c-value and potentially karyotype for this species, or closely related species.

Are sufficient details of methods and materials provided to allow replication by others?

Yes

Is the rationale for creating the dataset(s) clearly described?

Yes

Are the datasets clearly presented in a useable and accessible format?

Yes

Are the protocols appropriate and is the work technically sound?

Yes

Reviewer Expertise:

Zoology, phylogeny, evodevo, genomics

I confirm that I have read this submission and believe that I have an appropriate level of expertise to confirm that it is of an acceptable scientific standard.

# Peer review: Reviewer response for version 1

**DOI:** 10.21956/wellcomeopenres.21759.r62733

**Published:** 2023-07-17

**Authors:** Jian-Wen Qiu

**Affiliations:** 1 Department of Biology, Hong Kong Baptist University, Hong Kong, China

The paper is well-written. I have only some minor comments for the authors to consider during revision:

Background:

- The authors have provided some general information about the biology of the rag worm, but it would be helpful to also provide some background about the genomic and transcriptomic resources available for this species or even Errantia to justify this genome sequencing study.
- It is awkward to read when a sentence has three “feeding”: “feeding using a combination of deposit feeding and suspension feeding, the latter by using mucus nets”. Consider changing the first “feeding” to “obtaining food”.
- In “. (Devon & Severn IFCA, 2019; Olive, 1994; Watson et al., 2017)”, move “.” to the end of the sentence.

Sequencing:

- Some critical details of RNAseq should be added: paired-end,  ??? bp? Their use (presumably in annotation?) should be mentioned.

Are sufficient details of methods and materials provided to allow replication by others?

Partly

Is the rationale for creating the dataset(s) clearly described?

Partly

Are the datasets clearly presented in a useable and accessible format?

Yes

Are the protocols appropriate and is the work technically sound?

Yes

Reviewer Expertise:

Invertebrate biology and genome analysis

I confirm that I have read this submission and believe that I have an appropriate level of expertise to confirm that it is of an acceptable scientific standard.

# Peer review: Reviewer response for version 1

**DOI:** 10.21956/wellcomeopenres.21759.r62734

**Published:** 2023-07-17

**Authors:** José María Martín-Durán

**Affiliations:** 1 School of Biological and Behavioural Sciences, Queen Mary University of London, London, UK

This is a high-quality reference genome assembly for the annelid Alitta virens, a common invertebrate in the UK and European shores. The assembly will be a valuable resource for diverse research fields, from conservation and ecology to evolutionary and developmental biology.

 This genome note follows gold-standard practices, and I do not have any major comments. As a minor text point, I suggest changing A. virens to Alitta virens when starting a sentence.

Are sufficient details of methods and materials provided to allow replication by others?

Yes

Is the rationale for creating the dataset(s) clearly described?

Yes

Are the datasets clearly presented in a useable and accessible format?

Yes

Are the protocols appropriate and is the work technically sound?

Yes

Reviewer Expertise:

Annelid developmental biology, comparative genomics, evo-devo

I confirm that I have read this submission and believe that I have an appropriate level of expertise to confirm that it is of an acceptable scientific standard.
